# Evaluation of various deformable image registration algorithms for thoracic images

**DOI:** 10.1093/jrr/rrt093

**Published:** 2013-07-17

**Authors:** Noriyuki Kadoya, Yukio Fujita, Yoshiyuki Katsuta, Suguru Dobashi, Ken Takeda, Kazuma Kishi, Masaki Kubozono, Rei Umezawa, Toshiyuki Sugawara, Haruo Matsushita, Keiichi Jingu

**Affiliations:** 1 Department of Radiation Oncology, Tohoku University School of Medicine, 1-1 Seiryo-machi, Aoba-ku, Sendai 980-8574, Japan; 2 Department of Radiological Technology, School of Health Sciences, Faculty of Medicine, Tohoku University, 1-1 Seiryo-machi, Aoba-ku, Sendai 980-8574, Japan; 3 Radiation Technology, Tohoku University Hospital, 1-1 Seiryo-machi, Aoba-ku, Sendai 980-8574, Japan

**Keywords:** radiotherapy, deformable image registration, adaptive radiotherapy, image fusion

## Abstract

We evaluated the accuracy of one commercially available and three publicly available deformable image registration (DIR) algorithms for thoracic four-dimensional (4D) computed tomography (CT) images. Five patients with esophagus cancer were studied. Datasets of the five patients were provided by DIR-lab (dir-lab.com) and consisted of thoracic 4D CT images and a coordinate list of anatomical landmarks that had been manually identified. Expert landmark correspondence was used for evaluating DIR spatial accuracy. First, the manually measured displacement vector field (mDVF) was obtained from the coordinate list of anatomical landmarks. Then the automatically calculated displacement vector field (aDVF) was calculated by using the following four DIR algorithms: B-spine implemented in Velocity AI (Velocity Medical, Atlanta, GA, USA), free-form deformation (FFD), Horn–Schunk optical flow (OF) and Demons in DIRART of MATLAB software. Registration error is defined as the difference between mDVF and aDVF. The mean 3D registration errors were 2.7 ± 0.8 mm for B-spline, 3.6 ± 1.0 mm for FFD, 2.4 ± 0.9 mm for OF and 2.4 ± 1.2 mm for Demons. The results showed that reasonable accuracy was achieved in B-spline, OF and Demons, and that these algorithms have the potential to be used for 4D dose calculation, automatic image segmentation and 4D CT ventilation imaging in patients with thoracic cancer. However, for all algorithms, the accuracy might be improved by using the optimized parameter setting. Furthermore, for B-spline in Velocity AI, the 3D registration error was small with displacements of less than ∼10 mm, indicating that this software may be useful in this range of displacements.

## INTRODUCTION

There are several commercially or publicly available deformable image registration (DIR) algorithms that have been applied to multimodality image fusion, automatic image segmentation, four-dimensional (4D) image-guided radiotherapy and lung functional (ventilation) imaging [1–6]. However, there have been limited studies on geometric accuracy, and there has been no study using thoracic images with commercially available DIR algorithms. Recently, fully automatic DIR software (Velocity AI, Velocity Medical Solutions, Atlanta, GA, USA) has become commercially available [7].

It is necessary to verify the accuracy of commercially available automatic DIR software for use in a clinical setting. A number of reference standards have been utilized for validation of DIR software, including synthetically deformed images, high-contrast phantoms and expert-delineated control points [8–9]. While synthetic images and phantoms might provide useful qualitative evaluation of DIR performance characteristics, they lack sufficient realism to provide credible validation of registration spatial accuracy for 4D dose calculation, automatic segmentation and 4D computed tomography (CT) ventilation imaging in patients with thoracic cancer [10]. Therefore, the best reference standard is one derived from actual patient image data. The 4D CT images provided by DIR-lab are images of actual patient data including a large range of reference sample sizes, with equally varying spatial distributions. DIR-lab images have been proposed for characterization of DIR spatial accuracy performance [11].

Thus, in this study, we evaluated the accuracy of one commercially available and three publicly available DIR algorithms using thoracic 4D CT images and anatomical landmark sets.

## MATERIALS AND METHODS

### Commercially available DIR software (Velocity AI)

Velocity AI automatic DIR software was evaluated. B-spline and Demons algorithms were implemented in the software. Demons algorithms were first implemented in Velocity AI (ver. 2.7.0) but in the latest version (ver. 2.8.1) B-spline was only implemented. Therefore, the B-spline algorithm was used in this study. The Velocity's B-spline model was based on Mattes formulation of mutual information with some proprietary information that the vendor cannot divulge. This similarity metric was chosen for its simplicity and efficiency [12–13]. The B-spline model defines the deformation only on a sparse lattice of nodes overlaid on the image, and the displacement at any voxel is obtained by interpolation from the closet lattice nodes. This B-spline model is based on the open source ITK toolkit [13]. The prototype of Velocity's B-spline used the setting of 50 histogram bins and 10 percentage samples for Mattes mutual information metric. The uniform B-spline at one resolution was used and the L-BFGS (limited-memory Broyden–Fletcher–Goldfarb–Shanno) optimizer was used to find the optimal node value, with a maximum of 100 iterations and a maximum of 20 corrections used as termination conditions for the optimization algorithm [14–15]. Information on the parameter setting in the current version of this software, which was used in this study, was not made available by the vendor; the parameter setting cannot be changed by the user. Two DIR strategies (Deformable and Deformable multipass) were implemented in Velocity AI. Deformable is an approach to deform one image in a single stage, the resolution of which is determined by the user. Deformable multipass is an approach to perform DIR sequentially from low resolution to high resolution. After registration has been completed in one image resolution stage, the result is used as the initial condition for the next image registration stage. These resolutions for each stage are automatically determined. Since Deformable multipass is recommended by the vendor for use in a clinical setting and also for the purpose of reducing data complexity during DIR, this approach was used in this study.

### Publicly available DIR algorithms (DIRART)

To compare the results of Velocity AI with results obtained by other DIR algorithms, free-form deformation (FFD), Horn–Schunk optical flow (OF) and Demons in DIRART of MATLAB software were used [16–19]. FFD in DIRART is fast free-form deformation, which minimalizes an energy functional that combines both similarity and smoothness measures. By using calculus of variations, the minimization problem was represented as a set of non-linear elliptic partial differential equations (PDEs) [16]. A Gauss–Seidel finite difference scheme was used to iteratively solve the PDE [14]. OF and Demons are similar algorithms for intensity-based registration with the sum of square of intensity differences metric, and thus would not normally be considered good choices for multimodality image registration [17–18]. In this study, modified Demons included in DIRART was used. This algorithm is modified in the way that the gradient of the moving image is used of the gradient of the fixed image. In DIRART, multigrid and multiple pass approaches are used. Multigrid is almost equal to Deformable multipass in Velocity AI. The default setting in this software is a four-stage setting and was used in this study. The iteration numbers for each stage were 30 (Stage 1), 30 (Stage 2), 30 (Stage 3) and 40 (Stage 4). Stage 4 was the highest resolution. A multiple pass approach made it possible to perform registration in multiple passes, and for each pass, the registration was computed with a small number of iterations. Multiple passes with a small number of iterations for each pass would generate better results than one pass with a larger number of iterations. The numbers of passes for Stage 1, Stage 2, Stage 3 and Stage 4 were 2, 3, 4 and 5, respectively. The stop condition for iteration was set to 0.002. Iteration will stop if adjustment of the motion field is less than this condition. The stop condition for the multiple passing was set to 0.001. Pass will stop if the adjustment of motion field is less than this condition. These values were in units of voxel in 3D. After each pass the deformation vector field computed by the pass could be smoothed by a Gaussian low-pass fitter. The sigma of the Gaussian low-pass filter was set to 0.5 in voxel size. If the value was 1, then no smoothing was performed. The α^2^ parameter in the OF and the Gaussian low-pass filter window size in Demons were designed to control the smoothing operation in the iteration. The two values of α^2^ and the Gaussian low-pass filter window size were set to 0.2 and 3 in voxel size, respectively. To ensure variation in DIR spatial accuracy performance, no attempts were made to optimize individual case registrations.

### CT data and landmark point sets

**Fig. 1.**
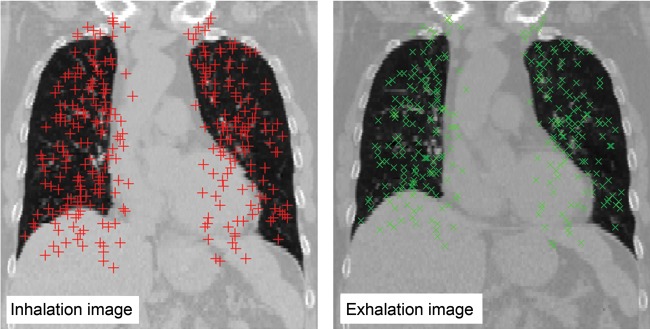
An example of inhale and exhale imaging with 300 landmark pairs.

Five patients with esophagus cancer were studied. Datasets of the five patients were provided by DIR-lab (www.DIR-lab.com) and consisted of thoracic 4D CT images acquired as part of the standard treatment planning process at 2.5-mm slice spacing with a General Electric Discovery ST PET/CT scanner (GE Medical Systems, Waukesha, WI) and a coordinate list of anatomical landmarks that had been manually identified and registered by an expert in thoracic imaging [11]. Extreme inhale and exhale phases of the 4D CT image sets were utilized in this study. Table 1 summarizes the CT image and reference landmark characteristics. Each image was cropped to include the entire rib cage and content subsampled 256 × 256 voxels. Final in-plane voxel dimensions ranged from 0.97 × 0.97 to 1.16 × 1.16 mm. For all cases, the ﬁnal image slice thickness was 2.5 mm. Regarding the landmark point sets, the original landmark points used in the paper by Castillo contained more than 1000 landmarks. The data of DIR-lab were 300 landmarks from original landmarks [20]. Expert landmark correspondence was used for evaluating DIR spatial accuracy. Figure 1 shows inhale and exhale images with the 300 landmark pairs.

**Table 1.** CT image and reference landmark characteristics

		Displacement	Displacement	Image	Voxel
Case	Number of landmarks	mean (mm)	SD (mm)	dimension	size (mm)
1	300	3.89	2.78	256 × 256 × 94	0.97 × 0.97 × 2.5
2	300	4.34	3.90	256 × 256 × 112	1.16 × 1.16 × 2.5
3	300	6.94	4.05	256 × 256 × 104	1.15 × 1.15 × 2.5
4	300	9.83	4.86	256 × 256 × 99	1.13 × 1.13 × 2.5
5	300	7.48	5.51	256 × 256 × 106	1.10 × 1.10 × 2.5

SD = standard deviation.

### Evaluation of DIR

The goal of DIR is to find a point-to-point correspondence from one image set to another. First, the manually measured displacement vector field (mDVF) was calculated by using the coordinate list. Then the automatically calculated displacement vector field (aDVF) was calculated by using DIR software. Registration errors between mDVF and aDVF were calculated. Mean registration errors were also determined over the combined set of landmarks for all cases. Additionally, 3D registration error was quantified via the distance between the target and the source landmark.

### Statistical methods

The registration errors were quantified as the differences between the calculated and manually measured landmark displacements.

Tukey's honestly significant difference test was used to compare the mean registration errors between the two DIR algorithms selected from four DIR algorithms and across all five cases. All tests were two-sided with *P*-values < 0.05 considered significant. Statistical analysis was performed with JMP version 9.0.2 (SAS Institute, Cary, NC).

## RESULTS

**Fig. 2.**
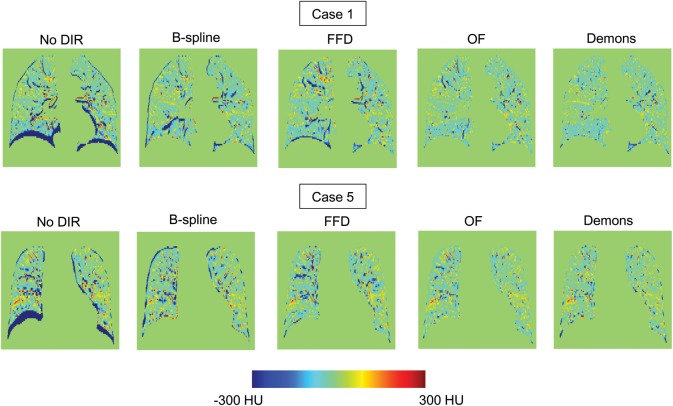
Difference images for registration of inhalation-to-exhalation 4D CT volumes for Case 1 and Case 5. No DIR = no deformable image registration, B-spline = deformable image registration with B-spline, FFD = deformable image registration with free-form deformation, OF = deformable image registration with Horn–Schunk optical flow, Demons = deformable image registration with Demons.

Table 2 summarizes the accuracy of each DIR algorithm for the five cases. Mean (SD) 3D registration errors ranged from 1.84 (0.97) to 3.72 (3.17) mm for B-spline, 2.49 (1.21) to 4.52 (3.45) for FFD, 1.42 (0.92) to 3.40 (2.93) for OF and 1.40 (0.96) to 4.39 (4.23) for Demons. Over the cumulative set of 1500 landmark pairs, 3D registration errors were 2.70 (2.24) for B-spline, 3.64 (2.8) for FFD, 2.40 (2.04) for OF, and 2.42 (2.54) for Demons. No significant difference was found between OF and Demons (*P*-value: 0.99). However, differences between the other two combinations were significant (*P* value < 0.05). The overall mean right–left (RL) and anterior–posterior (AP) component errors were each <1 mm for B-spline and OF, whereas they were >1 mm for FFD and Demons. The largest improvement in overall component registration error occurred in the superior–inferior (SI) direction (B-spline: 1.88 (2.12), FFD: 2.84 (2.87), OF: 1.64 (1.91), Demons: 1.57 (2.15)), and the error for B-spline was greater than that for OF and Demons. Figure 2 shows the difference images for registration of inhalation to exhalation 4D CT volumes for Case 1 and Case 5. For both cases, there are a few large differences in images by B-spline and FFD but only a few differences in images by OF and Demons.

**Table 2.** DIR registration accuracy

Case/Algorithm	RL		AP		SI		3D	
**Case 1;**								
B-spline (Velocity)	0.66	(0.49)	0.67	(0.60)	1.34	(1.03)	1.84	(0.97)
FFD (DIRART)	0.78	(0.71)	0.93	(0.70)	1.69	(1.34)	2.49	(1.21)
OF (DIRART)	0.63	(0.50)	0.56	(0.53)	1.10	(0.87)	1.55	(0.88)
Demons (DIRART)	0.46	(0.42)	0.52	(0.49)	1.05	(0.94)	1.40	(0.96)
No DIR	0.59	(0.59)	0.69	(0.80)	3.56	(2.90)	3.89	(2.78)
**Case 2**								
B-spline (Velocity)	0.62	(0.51)	0.72	(0.80)	1.41	(1.47)	1.90	(1.54)
FFD (DIRART)	0.78	(0.69)	0.97	(0.85)	2.04	(1.92)	2.66	(1.87)
OF (DIRART)	0.61	(0.54)	0.60	(0.61)	0.90	(0.81)	1.42	(0.92)
Demons (DIRART)	0.79	(0.87)	0.98	(1.01)	1.45	(1.61)	2.12	(1.89)
No DIR	0.71	(0.80)	0.70	(0.85)	3.78	(4.17)	4.34	(3.90)
**Case 3**								
B-spline (Velocity)	0.90	(0.79)	1.11	(1.24)	2.01	(1.74)	2.80	(1.84)
FFD (DIRART)	1.42	(1.04)	1.42	(1.19)	3.50	(3.15)	4.42	(3.03)
OF (DIRART)	1.20	(0.98)	1.01	(0.89)	1.99	(1.75)	2.84	(1.76)
Demons (DIRART)	1.17	(1.39)	0.89	(0.87)	1.24	(1.07)	2.22	(1.61)
No DIR	1.23	(1.07)	1.35	(1.32)	6.33	(4.28)	6.94	(4.05)
**Case 4**								
B-spline (Velocity)	0.89	(0.85)	1.26	(1.29)	3.06	(3.12)	3.72	(3.17)
FFD (DIRART)	1.21	(1.13)	1.11	(1.01)	3.82	(3.57)	4.52	(3.45)
OF (DIRART)	1.44	(1.35)	0.97	(0.93)	2.43	(2.92)	3.40	(2.93)
Demons (DIRART)	1.91	(2.00)	1.68	(1.85)	2.89	(3.86)	4.39	(4.23)
No DIR	1.01	(1.30)	1.42	(1.14)	9.39	(5.11)	9.83	(4.86)
**Case 5**								
B-spline (Velocity)	1.01	(0.94)	1.13	(1.37)	2.45	(2.29)	3.24	(2.40)
FFD (DIRART)	1.19	(1.03)	1.46	(1.39)	3.15	(3.09)	4.09	(3.06)
OF (DIRART)	0.92	(0.90)	1.27	(1.39)	1.79	(1.92)	2.77	(2.11)
Demons (DIRART)	0.75	(0.81)	0.92	(1.08)	1.21	(1.14)	1.97	(1.45)
No DIR	0.90	(1.03)	1.70	(1.67)	6.68	(5.85)	7.48	(5.51)
**Combined**								
B-spline (Velocity)	0.82	(0.75)	0.98	(1.13)	1.88	(2.12)	2.70	(2.24)
FFD (DIRART)	1.14	(0.96)	1.18	(1.08)	2.84	(2.87)	3.64	(2.80)
OF (DIRART)	0.96	(0.96)	0.88	(0.96)	1.64	(1.91)	2.40	(2.04)
Demons (DIRART)	1.02	(1.32)	1.00	(1.21)	1.57	(2.15)	2.42	(2.54)
No DIR	0.88	(1.01)	1.17	(1.27)	5.95	(5.04)	6.50	(4.83)

Quantitative evaluation of DIR spatial accuracy was performed for each algorithm using large samples of expert-determined landmark feature pairs as the reference. Mean 3D and component registration errors are shown with standard deviation for each case. All magnitudes are expressed in mm. LR = left–right, AP = anterior–posterior, SI = superior–inferior, 3D = three–dimensional; FFD = free-form deformation; OF = Horn–Schunk optical flow.

**Fig. 3.**
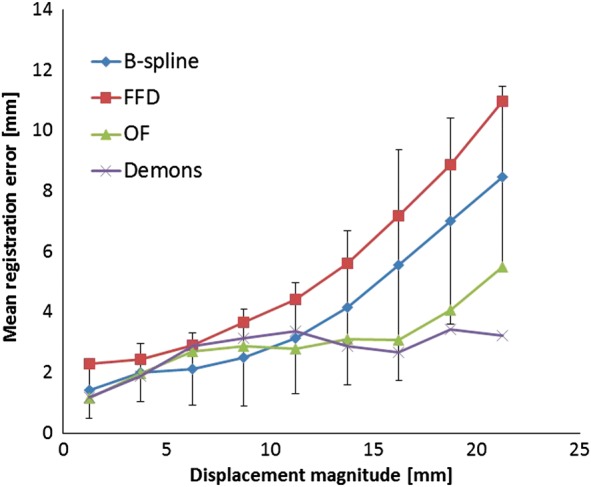
Registration error versus displacement magnitude. Only error bar (standard deviation) of B-spline is shown.

Figure 3 shows the results of 3D registration error versus landmark displacement magnitude over the set of reference point pairs for the four algorithms. Displacement magnitudes were binned into 2.5-mm increments. The average 3D errors with standard deviation for 3.75, 8.75, 13.75 and 18.75 mm motion distance magnitudes were 2.01 (0.97), 2.50 (1.60), 4.15 (2.54) and 7.01 (3.41) for B-spline, 2.44 (1.19), 3.65 (2.44), 5.62 (3.45) and 8.88 (2.77) for FFD, 1.97 (1.12), 2.88 (2.10), 3.09 (2.66) and 4.08 (3.56) for OF, and 1.90 (1.52), 3.13 (3.12), 2.87 (3.31) and 3.43 (3.78) for Demons, respectively. The figure shows that the behavior of OF and Demons 3D registration errors over the range of displacement magnitudes was more consistent than that of B-spline and FFD.

## DISCUSSION

Expert-determined sets of anatomical landmark feature pairs have become a common utility for evaluating DIR spatial accuracy, particularly in the context of clinically acquired thoracic images [21–23]. However, variability among reference datasets, particularly with regard to both the quantity and spatial uniformity of selected landmark features, can potentially yield numerical results that are misrepresentations of the true spatial accuracy, from which erroneous conclusions can be drawn with regard to the relative performance characteristics of multiple algorithms or implementations. Thus, in this study, we evaluated DIR spatial accuracy using expert-determined landmark features that are publically available on the Internet at the DIR-lab website (http://www.dir-lab.com). This dataset consists of 4D CT image sets and a large number of corresponding manually identified landmark points (300 pairs/case) between maximum phases.

Quantitative evaluations for the five cases are summarized in Table 2. Brock *et al*. assessed the accuracy of DIR for 12 groups for lung 4D CT and showed that all algorithms performed well for lung 4D CT, with mean absolute errors of ≤ 2.5 mm (slice thickness of the image set) in each direction [24].

**Fig. 4.**
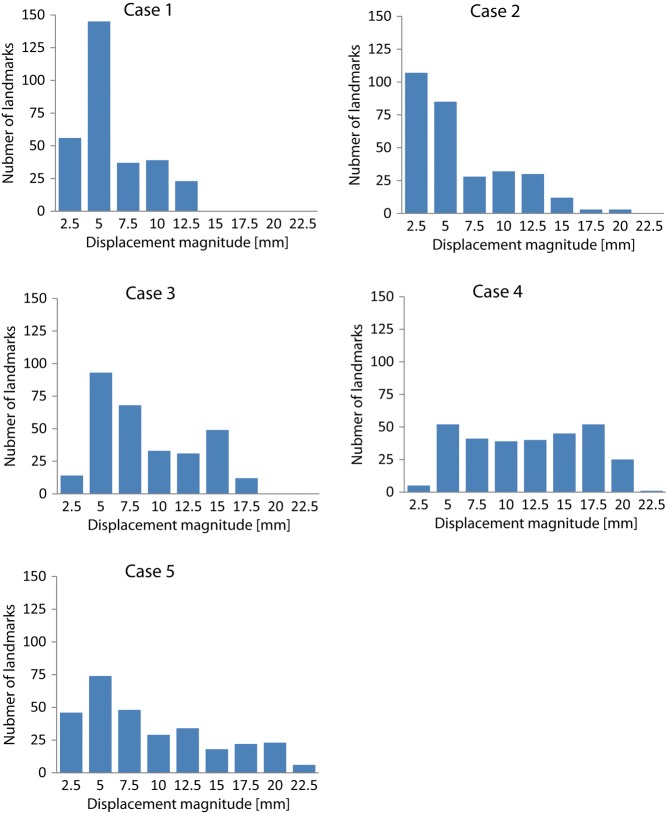
Histograms of the displacement magnitude of landmark pairs for each case.

The mean registration error in each direction in all cases, except for FFD (mean registration error in SI direction = 2.84), was <2.5 mm, which was the slice thickness of CT images. Gu *et al*. obtained the 3D registration error using the same five sets of thoracic images with various Demons algorithms and showed that the mean 3D registration error (standard deviation) for all cases was 1.57 mm (1.54 mm) [25]. Castillo *et al.* reported that the mean registration error for Cases 1, 2 and 5 by OF was 1.68 mm, and their data are comparable to our data calculated by 3D registration errors for Case 1, Case 2 and Case 5 in Table 2. (1.91 mm) [21]. Detailed analysis of Case 4 and Case 5 showed that the 3D registration errors for all algorithms were greater than those for other cases. The reason for this was that Case 4 and Case 5 had a higher percentage of large displacement magnitude of landmarks than did other cases, as shown in Fig. 4. That is, as shown in Fig. 3, a large displacement of landmarks gave rise to a large registration error.

In terms of reasonable comparison of the algorithms, the present study has several limitations. The DIR parameters in Velocity AI cannot be changed by the user, whereas those in DIRART can be changed by the user. On the other hand, Velocity AI is commercial software for clinical use, and its setting of parameters can be determined to achieve both speed and accuracy. Thus, the different parameter settings for each software may have caused a difference in the accuracy of DIR. Moreover, Demons and OF are monomodel registrations, considering images from one modality (i.e. CT–CT). B-spline with a similarity metric of mutual information is a multimodel registration, considering images from multiple modalities (i.e. CT–positron emission tomography). It was reasonable to use the sum of squared differences as a similarity metric in B-spline for comparison of Demons or OF with B-spline. Furthermore, results in this study may or may not indicate the maximum performance of each DIR algorithm. We should optimize the parameters for each DIR algorithm, if we know the maximum performance of each DIR algorithm. However, it is difficult and it takes a long time to determine the best parameters for each algorithm. Further research is thus needed to clarify this issue.

The fundamental framework for image registration generally requires four steps: namely, it requires a similarity metric (such as Mutual information), an interpolator (which defines how voxels get sampled during the registration process), a transformation (which specifies how a volume can change during the various steps in the optimization process, such as rigidly, affinely, deformably), and lastly the optimizer. The optimizer strives to find the best possible solution that registers the two volumes by marching over a small subset of the solution space. It does this by comparing the answers given by the similarity metric for the evaluated transformed spaces. Kashani *et al*. showed that different implementations, different users, and different parameter settings for the same type of registration can result in different accuracies, suggesting the need for careful assessment of each implementation as well as standards for user-defined parameters or automation of the registration process [26].

Next, as for the results of 3D registration error versus landmark displacement magnitude, the behavior of OF and Demons 3D registration errors over the range of displacement magnitudes was relatively consistent. As mentioned before, these two algorithms are monomodel registration algorithms. OF and Demons may have high DIR accuracy, even if displacement magnitudes are large. In B-Spline in Velocity AI, we tested only Deformable multipass. We may be able to obtain a higher level of accuracy by using Deformable several times with optimized parameters or by using Deformable with manually set high resolution, because the resolution of Deformable can be set by the user and optimized for each patient. Whether performing Deformable several times with optimized parameters manually improves DIR accuracy remains unclear. Further research is thus needed to clarify this issue. In B-spline in Velocity AI, the registration error was small with displacements of less than ∼ 10 mm. This result indicated that the parameter setting in Velocity AI might be optimized to obtain a high level of accuracy in the range of displacements < 10 mm, which is the range of displacements in usual clinical situations. However, Seppenwoolde *et al*. reported that the average amplitude of the tumor was greatest (12 mm) in the cranial–caudal direction for tumors situated in lower lobes, and that maximum amplitude of the tumor was 24.6 mm in the cranial–caudal direction [27]. That is, the registration error may be larger in lower lobes than in middle or upper lobes when DIR is performed for the whole lung to calculate 4D radiation doses [28–29]. The grid resolution of B-spline has a significant effect on registration error. A denser grid would result in improved registration accuracy at the cost of increased computational expense. Thus, if higher grid density can be used, the registration accuracy of B-spline may be better.

## CONCLUSION

We evaluated DIR algorithms including the DIR algorithm implemented in the commercial auto DIR system using patient CT images with a large number of anatomical landmark sets. The results showed that reasonable accuracy was achieved in B-spline, OF and Demons, and that these algorithms have the potential to be used in a clinical setting. However, for all algorithms, the accuracy might be improved by using the optimized parameter setting. Furthermore, for B-spline in Velocity AI, the registration error was small with displacements of less than ∼ 10 mm, indicating that this software may be useful in this range of displacements.

## FUNDING

This work was supported by JSPS-in-Aid for Young Scientists (B) (24791268).

## References

[1] Kessler M-L (2006). Image registration and data fusion in radiation therapy. Br J Radio.

[2] Ragan D, Starckschall G, McNutt T (2005). Semiautomated four-dimensional computed tomography segmentation using deformable models. Med Phys.

[3] Keall P (2004). 4-dimensional computed tomography imaging and treatment planning. Semin Radiat Oncol.

[4] Fallone B-G, Rivest D-R, Riauka T-A (2009). Assessment of a commercially available automatic deformable registration system. J Med Phys.

[5] Wu Z, Rietzel E, Boldea V (2008). Evaluation of deformable registration of patient lung 4DCT with subanatomical region segmentations. Med Phys.

[6] Yamamoto T, Kabus S, Klinder T (2011). Investigation of four-dimensional computed tomography-based pulmonary ventilation imaging in patients with emphysematous lung regions. Phys Med Biol.

[7] Velocity AI Velocity Medical Solutions. http://www.velocitymedical.com.

[8] Wang H, Dong L, O'Daniel J (2005). Validation of an accelerated demons algorithm for deformable image registration in radiation therapy. Phys Med Biol.

[9] Brock K-K, Nichol A-M, Menard C (2008). Accuracy and sensitivity of finite element model-based deformable registration of the prostate. Med Phys.

[10] Fitzpatrick J-M, Neuman MR, Hajnal J, Hill DL, Hawkes D (2001). Detecting Failure, Assessing Success. Medical Image Registration.

[11] DIR-lab http://www.dir-lab.com/ReferenceData.html.

[12] Mattes D, Haynor D-R, Vesselle H (2003). PET-CT image registration in the chest using free-form deformation. IEEE Trans Med Imaging.

[13] Ibanez L and Schroeder W *et al.* (2003). The ITK Software Guide.. Kitware Inc.

[14] Liu D-C, Nocedal J (1989). On the limited memory BFGS method for large scale optimization. Math Prog.

[15] Lawson J-D, Schreibmann E, Jani A-B (2007). Quantitative evaluation of a cone-beam computed tomography-planning computed tomography deformable image registration method for adaptive radiation therapy. J Appl Clin Med Phys.

[16] Lu W, Chen M-L, Olivera G-H (2004). Fast free-form deformable registration via calculus of variations. Phys Med Biol.

[17] Horn B-K, Schunck B-G (1981). Determining optical flow. Artif Intell.

[18] Thirion J-P (1998). Image matching as a diffusion process: an analogy with Maxwell's demons. Med Image Anal.

[19] Yang D, Brame S, Naga I-E (2011). DIRART—a software suite for deformable image registration and adaptive radiotherapy research. Med Phys.

[20] Castillo R, Castillo E, Guerra R (2009). A framework for evaluation of deformable image registration spatial accuracy using large landmark point sets. Phys Med Biol.

[21] Castillo E, Castillo R, Josue M (2010). Four-dimensional deformable image registration using trajectory modeling. Phys Med Biol.

[22] Brock K-K, Sharpe M-B, Dawson L-A (2005). Accuracy of finite element model-based multi-organ deformable image registration. Med Phys.

[23] Castillo E, Castillo R, Zhang Y (2009). Compressible image registration for thoracic computed tomography images. J Med Biol Eng.

[24] Brock K-K, Deformable Registration Accuracy Consortium (2010). Results of a multi-institution deformable registration accuracy study (MIDRAS). Int J Radiat Oncol Biol Phys.

[25] Gu X, Pan H, Liang Y (2010). Implementation and evaluation of various demons deformable image registration algorithms on a GPU. Phys Med Biol.

[26] Kashani R, Hub M, Balter J-M (2008). Objective assessment of deformable image registration in radiotherapy: a multi-institution study. Med Phys.

[27] Seppenwoolde Y, Shirato H, Kitamura K (2002). Precise and real-time measurement of 3D tumor motion in lung due to breathing and heartbeat, measured during radiotherapy. Int J Radiat Oncol Biol Phys.

[28] Paganetti H, Jiang H, Adams J-A (2004). Monte Carlo simulations with time-dependent geometries to investigate effects of organ motion with high temporal resolution. Int J Radiat Onocol Biol Phys.

[29] Chan M-K, Kwong D-L, Ng S-C (2012). Investigation of four-dimensional (4D) Monte Carlo calculation in real-time tumor tracking stereotactic body radiotherapy for lung cancers. Med Phys.

